# *Lycium barbarum* Polysaccharides Protect Rat Corneal Epithelial Cells against Ultraviolet B-Induced Apoptosis by Attenuating the Mitochondrial Pathway and Inhibiting JNK Phosphorylation

**DOI:** 10.1155/2017/5806832

**Published:** 2017-07-17

**Authors:** Shaobo Du, Biao Han, Kang Li, Xuan Zhang, Xueli Sha, Lan Gao

**Affiliations:** ^1^School of Life Sciences, Lanzhou University, Lanzhou 730000, China; ^2^Department of Thoracic Surgery, The First Hospital of Lanzhou University, Lanzhou 730000, China

## Abstract

*Lycium barbarum* polysaccharides (LBPs) have been shown to play a key role in protecting the eyes by reducing the apoptosis induced by certain types of damage. However, it is not known whether LBPs can protect damaged corneal cells from apoptosis. Moreover, no reports have focused on the role of LBPs in guarding against ultraviolet B- (UVB-) induced apoptosis. The present study aimed to investigate the protective effect and underlying mechanism of LBPs against UVB-induced apoptosis in rat corneal epithelial (RCE) cells. The results showed that LBPs significantly prevented the loss of cell viability and inhibited cell apoptosis induced by UVB in RCE cells. LBPs also inhibited UVB-induced loss of mitochondrial membrane potential, downregulation of* Bcl-2*, and upregulation of* Bax* and caspase-3. Finally, LBPs attenuated the phosphorylation of c-Jun NH_2_-terminal kinase (JNK) triggered by UVB. In summary, LBPs protect RCE cells against UVB-induced damage and apoptosis, and the underlying mechanism involves the attenuation of the mitochondrial apoptosis pathway and the inhibition of JNK phosphorylation.

## 1. Introduction

Like skin and its epithelial cells, known as keratinocytes, the eye and its corneal epithelial cells are exposed to ultraviolet (UV) irradiation directly and constantly. Among all UV wavelengths, the cornea is most sensitive to UVB rays, with 92% of UVB irradiation absorbed by the cornea to protect the inner eye [1]. Excess UVB irradiation may induce edema, photokeratitis, photophthalmia, and epithelial damage in the cornea [2, 3]. UVB irradiation has been shown to induce corneal epithelial cell damage, including decreased cellular viability, an increased number of apoptotic cells, and degradation of nuclei and mitochondria [4]. UVB irradiation-induced apoptosis has been associated with increased expression of* Bax*, cytochrome c, and caspase-3, as well as the loss of mitochondrial membrane potential (MMP) [4–7]. These features are characteristic of the intrinsic pathway of apoptosis, also known as the mitochondrial pathway [8]. Moreover, UVB irradiation has been found to activate molecules in the mitogen-activated protein kinase (MAPK) pathway, such as c-Jun NH_2_-terminal kinase (JNK), in corneal epithelial cells [9, 10].

*Lycium barbarum* (also known as wolfberry or Gou Qi Zi) is a traditional Chinese herbal medicine (fructus lycii) that has been used for thousands of years for nourishing the liver and kidney, helping to rebalance the “yin” and “yang” in the body, strengthening eyesight, and protecting the eyes [11, 12]. It is used for multiple pharmacological and biological benefits, including neuroprotective, antioxidant, antiaging, and cytoprotective effects [13].

*L. barbarum* polysaccharides (LBPs) are the main bioactive components of* L. barbarum*. Their molecular weights range from 24 to 241 kDa, and they are mainly composed of 6 types of monosaccharides: arabinose, glucose, galactose, mannose, xylose, and rhamnose [14]. Many studies have indicated that LBPs play an important role in eye protection and neuroprotection [15, 16]. For example, LBPs have been shown to protect retinal ganglion cells (RGCs) in chronic ocular hypertension [17], reduce secondary degeneration in the retina after partial optic nerve transection [18], and protect neurons against beta-amyloid peptide neurotoxicity in Alzheimer's disease [19]. The role of LBPs in protecting against corneal damage, however, remains unclear. In addition, it has been demonstrated that LBPs can reduce the apoptosis induced by some types of damage by attenuating the mitochondrial pathway. This includes the suppression of* Bax* and caspase-3 overexpression and inhibition of decreases in* Bcl-2* expression and MMP in neurons and spermatogenic cells [20, 21].

Prior studies on the protective effects of LBPs against apoptosis in eye cells demonstrated that LBPs can protect lens epithelial cells and retinal pigment epithelial cells from oxidative stress-induced apoptosis [13, 22] and photoreceptor cells from N-methyl-N-nitrosourea induced apoptosis [23]. However, it is not known whether LBPs can protect damaged corneal cells from apoptosis. Moreover, no reports have focused on the role of LBPs in guarding against UVB-induced apoptosis. Therefore, the present study evaluated the ability of LBPs to protect rat corneal epithelial (RCE) cells against UVB-induced damage and apoptosis by analyzing the effects on cell viability and apoptosis in vitro. The underlying mechanism of this protection was then explored further.

## 2. Materials and Methods

### 2.1. Isolation of RCE Cells and Cell Culture

RCE cells were isolated from adult female Wistar rats of standard body weight (200 g ± 20 g), purchased from the Animal Facility of the Medical School of Lanzhou University. All procedures and protocols in this study conform to the institutional guidelines of Lanzhou University and were approved by the Animal Experiment Ethics Committee of Lanzhou University. Primary cells were isolated according to the methods of Sobolewska et al. [24] and Kim et al. [25], with some modifications. Briefly, rat eyes were sterilized with 75% ethyl alcohol and extirpated. They were then placed in sterile phosphate-buffered saline (PBS) supplemented with 1% (v/v) penicillin-streptomycin (100 U/mL and 100 *μ*g/mL, resp.; Sangon Biotech, Shanghai, China) in 1.5 mL centrifuge tubes and washed at least 3 times. Corneas were trimmed off along the corneal limbal rims, and excess sclera was removed with sterile ophthalmic scissors. Corneal endothelia and stromata were carefully peeled off using sterile thin-tipped surgical forceps under a stereoscopic dissection microscope (Zeiss, Oberkochen, Germany). Then, the residual corneal stroma was peeled off little by little again, and, finally, the straticulate epithelia were placed in 12-well cell culture plates. Next, 2 mL Dulbecco's modified Eagle's medium/nutrient mixture F-12 (D-MEM/F-12; pH 7.0–7.2; Gibco, Grand Island, NY, USA) supplemented with 10% (v/v) fetal bovine serum (Biological Industries, Kibbutz Beit Haemek, Israel) and 1% (v/v) penicillin-streptomycin (100 U/mL, 100 *μ*g/mL) was added. Epithelia were incubated in a 5% CO_2_ incubator (SANYO, Osaka, Japan) at 37°C. When the primary cells fell off, adhered to the plate, and grew to complete confluence, they were passaged in a culture bottle using trypsin. The passaged cells were cultured under the same conditions. We used cytokeratins 3 and 12, which are the specific marker proteins of corneal epithelium, to detect the purity of RCE cells by immunofluorescence. The purity of RCE cells was approximately 80%.

### 2.2. Preparation of LBP Cell Culture Medium

A stock solution of LBP (10 mg/mL in D-MEM/F-12) was prepared using LBP powder (polysaccharide content, 90.2%; Shaanxi Ciyuan Biotech Co., Ltd., Xi'an, China) [26]. For the experiments, the stock solution was diluted to final concentrations using D-MEM/F-12 to create LBP medium.

### 2.3. UVB Treatment Procedure

When cells had grown to 80–90% confluence in the culture bottle, they were digested, resuspended, and then seeded in 35 mm Petri dishes containing 2 mL medium. These were used in subsequent experiments after the cells grew to 80–90% confluence.

UVB irradiation was performed on RCE cells as described previously by Shi and Isseroff [27], with some modifications. Cells were washed with PBS once and then covered with a thin layer of PBS (900 *μ*L PBS in 35 mm Petri dishes), which had been preheated to 37°C before use. Culture dishes without lids were placed 12 cm below a UVB treatment lamp tube (Huaqiang Electronic Company, Nanjing, China) in a clean bench with regular air conditions. The UVB irradiance herein was 20 *μ*W/cm^2^, as measured by the UV-297 probe of an ultraviolet radiation meter. According to previous studies on UVB-induced apoptosis and damage in corneal epithelial cells [4, 6, 28], the radiant exposure in the present study was determined to be 144 mJ/cm^2^. The formula for radiant exposure is given by “$H=t\times E_{\ddot{e}}$,” where “*H*” is radiant exposure in J/cm^2^, “*t*” is time in s, and “$E_{\ddot{e}}$” is the measured irradiance in W/cm^2^ [4]. On the basis of this formula, the cells were irradiated for 120 min, after which PBS was removed, and medium was added to the dish for further cell culture.

### 2.4. Experimental Model

To evaluate the influence of LBPs on RCE cells, the cells were seeded in 35 mm Petri dishes. At 80% confluence, the growth medium was replaced by LBP medium, and the cells were treated with different concentrations (0, 0.05, 0.1, 0.5, 1, 5, or 10 mg/mL) of LBPs for 24 h [22]. Cell viability was then assessed.

**Figure 1:**
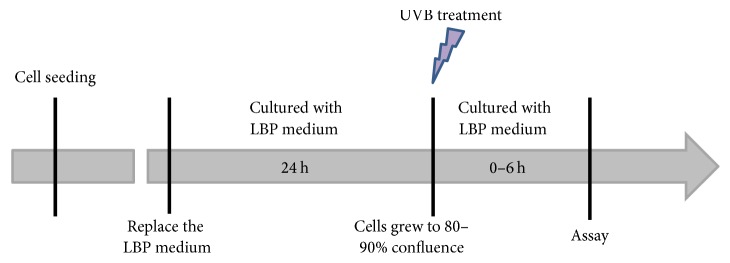
Schematic diagram showing the procedure for the experimental model corresponding to the LBP treatment group (UVB+/LBPs+).

To investigate the protective effects of LBPs against UVB-induced damage, the following procedure was performed (Figure 1). Cells were divided into 4 groups: sham irradiation control group (UVB−/LBPs−), LBP control group (UVB−/LBPs+), UVB irradiation group (UVB+/LBPs−), and LBP treatment group (UVB+/LBPs+). UVB treatment was performed in the UVB irradiation group and LBP treatment group as described. In the latter, the culture medium was replaced with LBP medium 24 h before UVB irradiation, and cells continued to be cultured in LBP medium after irradiation. The culture of the sham irradiation control group and LBP control group was identical to that of the UVB irradiation and LBP treatment groups, respectively, except for the absence of UVB irradiation.

### 2.5. Cell Viability Assay

The MTT assay is a standard method of assessing cell viability [29]. It is based on the conversion of 3-(4,5-dimethyl-2-thiazolyl)-2,5-diphenyl-2-H-tetrazolium bromide (MTT) to formazan by viable cells [30]. MTT (5 mg/mL, 10% volume of the medium; Beyotime, Shanghai, China) was added to cells in 35 mm Petri dishes and incubated for 4 h at 37°C in the dark. Then, the liquid was carefully removed by needle tubing, leaving the formazan product in the bottom. Next, 2 mL DMSO was added to the dishes, which were shaken slowly for 15 min to dissolve the formazan. The optical density (OD; 490 nm) was measured by a spectrophotometer (UNICO, Shanghai, China).

### 2.6. Cell Apoptosis Assay

To quantify cell apoptosis rates, annexin V-FITC and propidium iodide (PI) staining was used according to the manufacturer's protocol (Vazyme, Nanjing, China). The cells were collected, washed with PBS twice, and resuspended in 100 *μ*L binding buffer. Then, 5 *μ*L annexin V-FITC and 5 *μ*L PI staining solution was added to the buffer, and cells were incubated at room temperature for 10 min in the dark. After replenishing with 400 *μ*L binding buffer, cells were detected by a flow cytometer (LSRFortessa, BD Biosciences, Franklin Lakes, NJ, USA).

### 2.7. Observation of Cell Morphology

The cell morphology in each group was recorded using a differential interference contrast (DIC) microscope (Zeiss).

### 2.8. Mitochondrial Membrane Potential Assay

MMP was measured by rhodamine 123 (Rh123) staining [31]. Specifically, cells were collected and resuspended in PBS. They were then incubated with 1 *μ*g/mL Rh123 (Beyotime) at 37°C for 30 min in the dark. Rh123 fluorescence was subsequently monitored by flow cytometer (BD Biosciences).

### 2.9. Gene Expression Analysis

Total RNA was isolated using the TRIzol regent (Invitrogen, Carlsbad, CA, USA). Genomic DNA was eliminated and total RNA was reverse-transcribed to cDNA using the PrimeScript RT reagent kit (TaKaRa, Tokyo, Japan). To quantify relative gene expression, real-time PCR was performed using a SYBR Premix Ex Taq II Kit (TaKaRa) and a real-time PCR instrument (Agilent MX3005P, Palo Alto, CA, USA) according to the manufacturer's instructions. The 2^−ΔΔCt^ method [32] was used for calculating the expression levels of target genes. The primers used are shown in Table 1, and the endogenous control gene glyceraldehyde-3-phosphate dehydrogenase* (GAPDH)* was used as a reference.

### 2.10. Western Blotting

After treatment, cells were washed with cold PBS and then lysed adequately by RIPA buffer (Beyotime). Sodium dodecyl sulfate- (SDS-) polyacrylamide gel electrophoresis (PAGE) loading buffer was added to protein lysates, which were boiled and then clarified by centrifugation at 12,000 ×g for 10 min at 4°C. Next, the lysates were separated by 12% or 15% SDS-PAGE and transferred onto a polyvinylidene difluoride membrane. Membranes were blocked with 5% skim milk in Tris-buffered saline with Tween 20 and incubated overnight at 4°C with the following specific primary rabbit polyclonal antibodies: anti-caspase-3, 1 : 500; anti-JNK, 1 : 250; anti-p-JNK (phosphor-Thr183/Y185), 1 : 500; and anti-GAPDH, 1 : 1000 (ImmunoWay Biotechnology, Plano, TX, USA). Membranes were then incubated with horseradish peroxidase-conjugated secondary goat anti-rabbit antibody (1 : 5000; ImmunoWay Biotechnology). Immunoreactive proteins were visualized using enhanced chemiluminescence, and the optical density of each band was measured and quantified using ImageJ software (National Institutes of Health, Bethesda, MD, USA).

### 2.11. Statistical Analysis

Data were analyzed using SPSS 19.0 (IBM, Chicago, IL, USA). Values are presented as the mean ± standard deviation. One-way analysis of variance with the least significant difference test was used to make statistical comparisons between groups. Significance was determined at *P* < 0.05.

## 3. Results

### 3.1. LBPs Increased the Cell Viability of UVB-Irradiated RCE Cells

**Figure 2:**
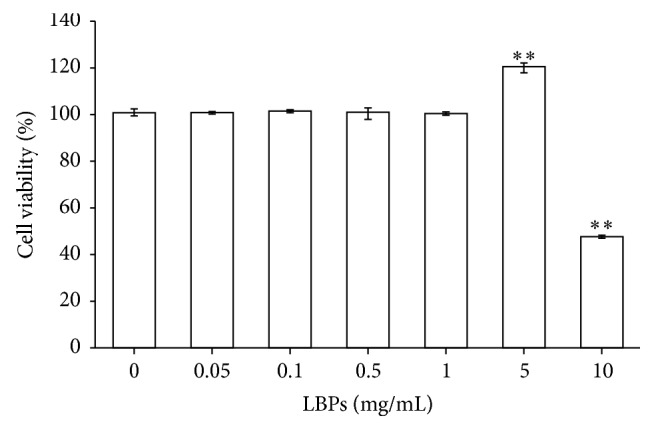
Effects of LBPs on the cell viability of RCE cells. Cells were incubated for 24 h in different concentrations of LBPs. Data are presented as mean ± standard deviation, *n* = 3. ^*∗∗*^*P* < 0.01 compared with 0 mg/mL LBPs.

RCE cells were incubated with different concentrations (0, 0.05, 0.1, 0.5, 1, 5, or 10 mg/mL) of LBPs for 24 h, and cell viabilities were analyzed by the MTT assay. We found that 0-1 mg/mL LBPs did not affect cell viability, while 5 mg/mL LBPs increased cell viability (Figure 2). In contrast, 10 mg/mL LBPs resulted in a significant reduction in cell viability owing to the presence of excess polysaccharides.

**Figure 3:**
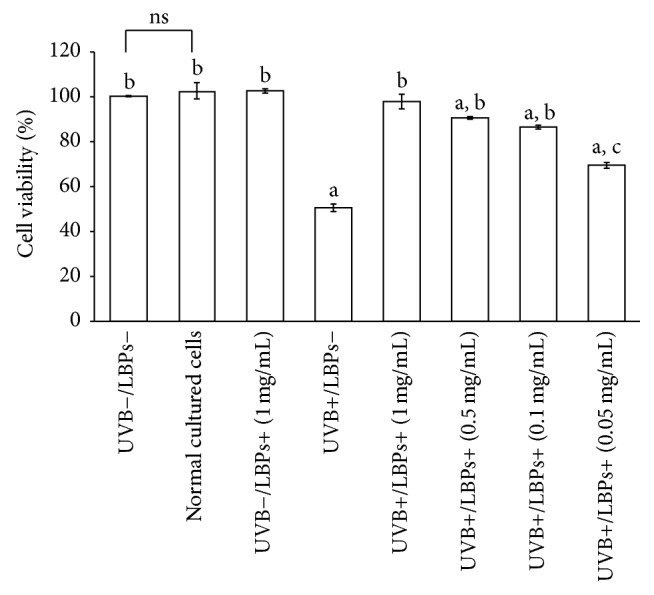
LBPs prevented the UVB-induced decline in cell viability in RCE cells. Data are presented as mean ± standard deviation, *n* = 3. ^a^*P* < 0.01 compared with the UVB−/LBPs− group; ^b^*P* < 0.01 compared with the UVB+/LBPs− group; ^c^*P* < 0.05 compared with the UVB+/LBPs− group. ns, no significant difference.

On the basis of these results, LBP concentrations of 0.05, 0.1, 0.5, and 1 mg/mL were selected for subsequent assays. To determine whether LBPs protect corneal epithelial cells against UVB-induced cell damage, cells were treated with one of these 4 concentrations of LBPs and irradiated with UVB. Cell viabilities were checked 6 h after UVB irradiation using the MTT assay. As shown in Figure 3, cell viability in the sham irradiation control group was not significantly different from that of the normal cultured cells. That is to say, sham irradiation did not change the viability of RCE cells. Furthermore, cell viability demonstrated an observable decline after irradiation with 144 mJ/cm^2^ UVB. LBP treatment at concentrations of 0.05–1 mg/mL, however, prevented a UVB-induced decline in cell viability. The optimal concentration of LBPs for this protective effect was 1 mg/mL.

### 3.2. LBPs Reduced UVB-Induced Apoptosis in RCE Cells

**Figure 4:**
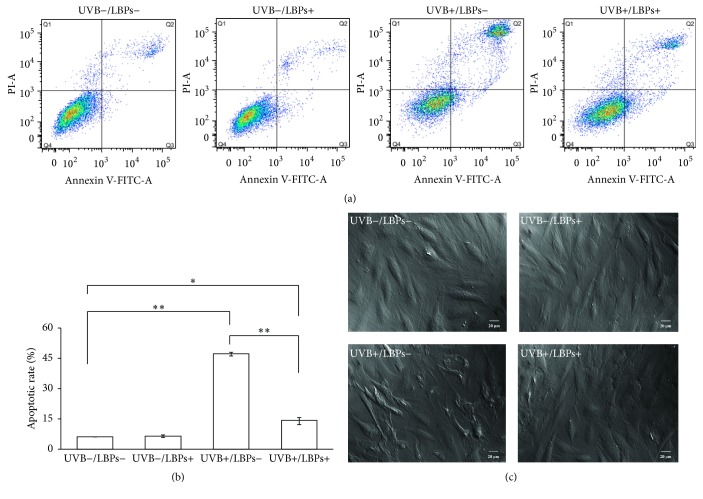
Inhibitory effect of 1 mg/mL LBPs on UVB-induced apoptosis in RCE cells. (a) Flow cytometry scatter plot, including UVB−/LBPs−, UVB−/LBPs+, UVB+/LBPs−, and UVB+/LBPs+ groups. Q1: dead cells; Q2: late apoptosis cells; Q3: early apoptosis cells; Q4: normal cells. (b) Histogram of the quantitative change in apoptotic rates. Apoptotic rate = early cellular apoptotic rate + late cellular apoptotic rate. Data are presented as mean ± standard deviation, *n* = 3. ^*∗*^*P* < 0.05 and ^*∗∗*^*P* < 0.01. (c) Changes in cellular morphology of RCE cells in each group.

On the basis of the results of the MTT assay, LBPs at a concentration of 1 mg/mL were used for subsequent experiments. Cells were divided into 4 groups (UVB−/LBPs−, UVB−/LBPs+, UVB+/LBPs−, and UVB+/LBPs+), and the procedure depicted in Figure 1 was performed. Rates of cell apoptosis were assessed 6 h after UVB irradiation using annexin V-FITC and PI staining and analyzed by flow cytometry. The cell apoptosis rate was calculated as a sum of the early cellular apoptotic rate and late cellular apoptotic rate. The results demonstrated that the apoptotic rate in the LBP control group (UVB−/LBPs+) was not significantly different from that in the sham irradiation control group (UVB−/LBPs−). However, the apoptotic rate in the LBP treatment group (UVB+/LBPs+) was 13.93%  ±  1.76%, which was significantly lower than the rate in the UVB irradiation group (UVB+/LBPs−; 47.06%  ±  1.83%) (Figures 4(a) and 4(b)).

Cell morphological changes were also observed 6 h after UVB irradiation using a DIC microscope. It was found that the cell morphology in the LBP control group (UVB−/LBPs+) did not significantly differ from that in the sham irradiation control group (UVB−/LBPs−). However, the cells appeared to have typical apoptotic morphology, including cell shrinkage, decline in cell adherence ability, cell membrane rupture, and nuclear condensation in the UVB irradiation group (UVB+/LBPs−). In the LBP treatment group (UVB+/LBPs+), the cell morphological changes were very little compared with the UVB−/LBPs− group (Figure 4(c)). Therefore, both the cell apoptotic rates and cell morphology demonstrated that treatment with 1 mg/mL LBPs appeared to lead to a remarkable reduction in UVB-induced apoptosis in RCE cells.

### 3.3. LBPs Prevented UVB-Induced Loss of MMP

**Figure 5:**
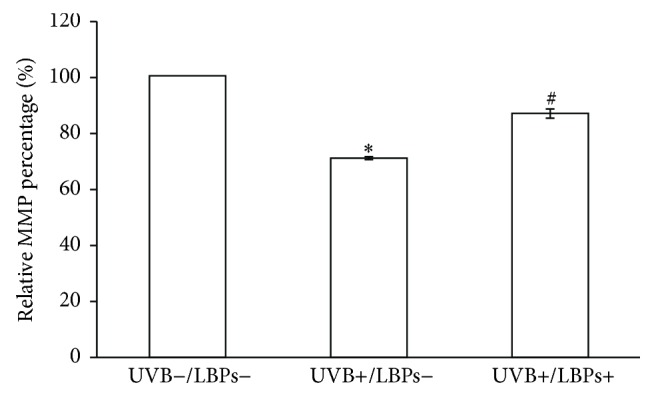
Relative fluorescence intensities of rhodamine 123, showing that LBPs prevent UVB-induced MMP loss. Data are presented as mean ± standard deviation, *n* = 3. ^*∗*^*P* < 0.05 compared with the UVB−/LBPs− group; ^#^*P* < 0.05 compared with the UVB+/LBPs− group.

To determine whether the LBP-induced reduction in apoptosis involved the mitochondrial pathway, cell MMPs were measured 3 h after irradiation. Because treatment with 1 mg/mL LBP alone did not affect cell apoptosis (Figure 4), this test included only 3 treatment groups: UVB−/LBPs−, UVB+/LBPs−, and UVB+/LBPs+. The results demonstrated that the MMP decreased to 70.57%  ±  0.97% of that of the UVB−/LBPs− group after UVB irradiation (UVB+/LBPs−), and it recovered to 86.59%  ±  1.62% of that of the UVB−/LBPs− group when the cells were protected with LBPs (UVB+/LBPs+) (Figure 5). Therefore, 1 mg/mL LBPs prevented UVB-induced loss of MMP. This suggests that LBPs inhibit UVB-induced apoptosis through the mitochondrial pathway.

### 3.4. LBPs Influenced the Expression of Bax, Bcl-2, and Caspase-3

**Figure 6:**
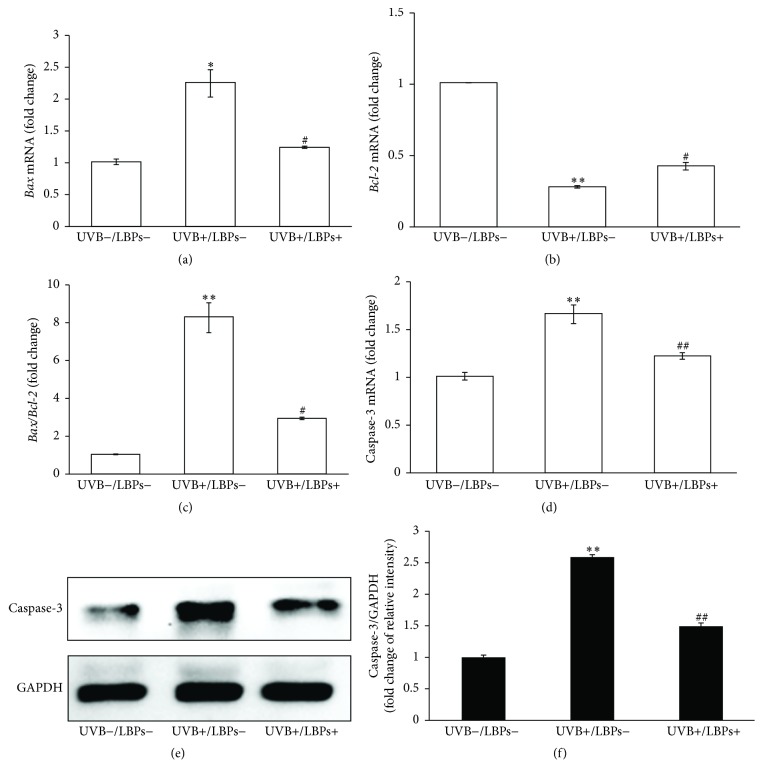
LBPs inhibit UVB-induced upregulation of* Bax* and caspase-3 and downregulation of* Bcl-2* in RCE cells. (a, b) Relative mRNA expression changes of* Bax* and* Bcl-2* in UVB−/LBPs−, UVB+/LBPs−, and UVB+/LBPs+ groups. (c) Fold changes in the* Bax*/*Bcl-2* ratio in each group. (d) Relative mRNA expression changes of caspase-3 in each group. (e) Changes in the protein levels of caspase-3 were confirmed by Western blotting. GAPDH was used as an endogenous control protein. (f) Fold changes in the relative optical intensities of caspase-3/GAPDH in each group. ^*∗*^*P* < 0.05 and ^*∗∗*^*P* < 0.01 compared with the UVB−/LBPs− group; ^#^*P* < 0.05 and ^##^*P* < 0.01 compared with the UVB+/LBPs− group; *n* = 3 in each group.

To further elucidate the mechanism of LBPs in reducing UVB-induced apoptosis, the mRNA expression levels of* Bax* and* Bcl-2*, 2 apoptosis-related factors that act in the mitochondrial pathway, were detected using q-PCR. We found that UVB upregulated* Bax* mRNA levels (2.25 ± 0.21-fold), downregulated* Bcl-2* mRNA levels (0.27 ± 0.01-fold), and significantly increased the* Bax*/*Bcl-2* ratio (8.26 ± 0.79-fold) compared with that in the sham irradiation control group (UVB−/LBPs−). However, these trends were significantly inhibited in the UVB+/LBPs+ group, limiting the increase in* Bax* to 1.23 ± 0.02-fold, the decrease in* Bcl-2* to 0.42 ± 0.03-fold, and the increase in the* Bax*/*Bcl-2* ratio to 2.91 ± 0.08-fold (Figures 6(a), 6(b), and 6(c)). These results further indicate that the protective effect of LBPs against UVB-induced apoptosis involves the mitochondrial pathway.

In addition, the expression of caspase-3, which is a downstream factor and executor of apoptosis, was measured at both the mRNA and protein levels 6 h after UVB irradiation using q-PCR and Western blotting, respectively. Results showed that caspase-3 was activated by UVB and that treatment with LBPs inhibited the expression of caspase-3 (Figures 6(d), 6(e), and 6(f)).

### 3.5. LBPs Attenuated the UVB-Induced Phosphorylation of JNK

**Figure 7:**
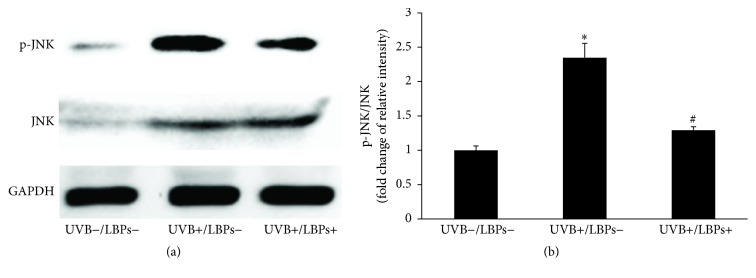
LBPs attenuate the phosphorylation of JNK triggered by UVB in RCE cells. (a) Both p-JNK and nonphosphorylated JNK were detected by Western blotting. GAPDH was used as an endogenous control protein. (b) Fold changes of the relative optical intensities of p-JNK/JNK. ^*∗*^*P* < 0.05 compared with the UVB−/LBPs− group; ^#^*P* < 0.05 compared with the UVB+/LBPs− group; *n* = 3 in each group.

To determine whether LBPs altered the above-mentioned apoptotic factors via the JNK pathway, the expression of the active, phosphorylated form of JNK (p-JNK) was determined by Western blotting. Results showed that p-JNK levels increased after UVB irradiation; however, JNK phosphorylation was inhibited when cells were treated with 1 mg/mL LBPs (Figure 7). Therefore, the JNK pathway appears to be involved in UVB-induced apoptosis, and LBPs inhibit apoptosis by preventing the phosphorylation of JNK.

## 4. Discussion and Conclusions

The cornea is directly exposed to solar UV irradiation. The corneal epithelium, as the outermost layer of the cornea and the first barrier of the eye, absorbs a large percentage of UV irradiation and protects the lens and retina from UV damage [33]. However, acute UV irradiation, especially UVB irradiation, of the cornea can induce corneal disease, such as photokeratitis, corneal haze, edema, and corneal cell apoptosis [3, 34, 35]. Therefore, therapeutic treatments for protecting the cornea against UV-induced damage are needed. It has been found that the success rate for the development of new medicinal agents via the synthetic route might be as low as 1/10,000, while the success rate in the search for new therapeutic moieties based on medicinal plants used in traditional medicine might be as high as 1/4 [36]. We therefore focused our study on LBPs, which are water-soluble glycoconjugates isolated from the aqueous extracts of* L. barbarum*. LBPs have been found to protect retinal cells and inhibit apoptosis induced by many factors to protect the nervous and reproductive systems [14]. However, the relationship between LBPs and UVB-induced corneal cell damage is not well understood. Therefore, the present study focused on the protective effect and underlying mechanism of LBPs against UVB-induced damage in RCE cells in vitro.

Thus far, it has been unclear whether LBPs alone had any effects on cell viability. A previous study showed that 0-1 mg/mL LBPs had no obvious effect on cell viability in rat adrenal pheochromocytoma cells (PC 12 cells) [37]. Another study showed that 0.05, 0.1, 0.2, 0.4, and 0.8 mg/mL LBPs exhibited no significant cytotoxicity in SV40 T antigen-transformed human lens epithelial (SRA01/04) cells; in fact, 0.2 and 0.4 mg/mL LBPs benefitted cell growth. However, cell cytotoxicity was detected at a concentration of 1.6 mg/mL LBPs [13]. In the present study, concentrations of 0–10 mg/mL LBPs were tested, and we found that 0-1 mg/mL LBPs had no effect on RCE cell viability; 5 mg/mL LBPs benefitted RCE cell growth; and 10 mg/mL LBPs was cytotoxic to RCE cells. These results therefore provide reference values for similar research in the future.

UVB irradiation reduces viability and increases apoptosis in corneal epithelial cells [28, 38]. Many in vitro studies have shown that LBPs prevent viability reductions induced by different factors in PC 12 cells [37], SRA01/04 cells [13], neuro-2a cells [39], and human retinal pigment epithelial cells [22]. LBPs have also been shown to exhibit cardioprotective effects against doxorubicin-induced cardiotoxicity [40], neuroprotective effects against beta-amyloid peptide and glutamate-induced neuronal injury [19, 41], and protective effects against bisphenol A-induced damage to the reproductive system [42] by reducing cell apoptosis. In the present study, we found that LBPs prevented UVB-induced decreases in cell viability and increases in cell apoptosis in RCE cells. Thus, LBPs exert a protective effect against UVB-induced damage of corneal epithelial cells by inhibiting cell apoptosis.

Apoptosis, also known as programmed cell death, is a regulated process that involves the activation of a series of molecular events [8]. Bcl-2 family proteins consist of both proapoptotic proteins, such as Bax, and antiapoptotic proteins, such as Bcl-2 [43]. Apoptosis is regulated by maintaining a balance between Bcl-2 and Bax, with increases in the Bax/Bcl-2 ratio promoting cell apoptosis [44]. It has been demonstrated that UVB irradiation can increase this ratio [5]. In addition, Bcl-2 family proteins act upstream of the mitochondria and govern mitochondrial membrane permeability [8]. While Bax normally resides in the cytosol, it translocates to the outer mitochondrial membrane in response to cell stimulation with lethal agents [45]. This results in an opening of the mitochondrial permeability transition pore, loss of MMP, and release of normally sequestered proapoptotic proteins, such as cytochrome c, from the intermembrane space into the cytosol [46]. Youn et al. found that the MMP was clearly reduced in human corneal epithelial (HCE) cells treated with UVB [4]. Once released, these proapoptotic proteins activate the caspase cascade and caspase-3, which is the final executor of apoptosis [47]. Activation of caspase-3 is a hallmark of apoptosis [48]. The above-mentioned process is known as the mitochondrial pathway of apoptosis [8]. In the present study, we found that UVB induced an increase in the Bax/Bcl-2 ratio, a decline in MMP, and the activation of caspase-3 in RCE cells, but LBPs prevented these events to different extents. Thus, LBPs inhibited UVB-induced apoptosis via the mitochondrial pathway in RCE cells. This is consistent with previous studies that have reported that LBPs reduce cell apoptosis by regulating the Bax/Bcl-2 ratio [20–22], MMP [21], and caspase expression [20, 23] in other experimental models in vivo and in vitro. Taken together, these results suggest that attenuating the mitochondrial apoptosis pathway is one of the major mechanisms underlying the protective effects of LBPs against cell damage.

The JNK cascade is part of the MAPK cascade, which is induced following cellular stress or cytokine signaling [49]. It has been shown that JNK can phosphorylate members of the Bcl-2 family of proteins, such as Bcl-2 and Bcl-xL, inactivating their antiapoptotic functions [49] and inducing the translocation of Bax to the outer mitochondrial membrane, which triggers the process that leads to the activation of caspase-3 [45]. Thus, the proapoptotic JNK cascade ultimately induces apoptosis via the mitochondrial pathway; this apoptotic pathway is also known as the JNK-Bax-caspase-3 pathway [45]. Previous studies have demonstrated UVB irradiation to activate JNK, as determined by an increase in the phosphorylated form of the enzyme in HCE cells and human keratinocytes [9, 50, 51]. Furthermore, LBPs have been shown to reduce glutamate- and homocysteine-induced phosphorylation of JNK in rat cortical neurons, resulting in a neuroprotective effect [41, 52]. In addition, LBPs can inhibit the JNK pathway to delay secondary degeneration of RGCs after partial optic nerve transection [18]. Our results indicated that UVB activates the phosphorylation of JNK in RCE cells, an effect that can be reduced by treatment with LBPs. These results demonstrate that LBPs attenuate the above-mentioned JNK-Bax-caspase-3 pathway to reduce UVB-induced apoptosis in RCE cells.

In summary, the present experiments demonstrated that LBPs have a protective effect against UVB-induced apoptosis in RCE cells. The underlying mechanism of this effect involves the attenuation of JNK phosphorylation, upregulation of* Bcl-2* expression, downregulation of* Bax* expression, inhibition of MMP loss, and downregulation of caspase-3 expression, all factors involved in the mitochondrial and JNK-Bax-caspase-3 pathway of apoptosis. To our knowledge, this is the first report to demonstrate the protective effects of LBPs against UV-induced damage and apoptosis in corneal cells. Given that LBPs are isolated from a traditional Chinese herbal medicinal formulation, they might exhibit fewer side effects. LBPs might thus be appropriate for use in anti-UV treatments to protect the eyes.

## Figures and Tables

**Table 1:** Primers of detected genes.

Gene	Accession number	Direction	Sequence	Product length (bp)
*Bax*	NM_017059.2	Forward	5′-CCACCAAGAAGCTGAGCGA-3′	127
		Reverse	5′-GCTGCCACACGGAAGAAGA-3′	
*Bcl-2*	NM_016993.1	Forward	5′-CTCTGTGGATGACTGAGTACCTG-3′	143
		Reverse	5′-GAGCAGCGTCTTCAGAGACAG-3′	
Caspase-3	NM_012922.2	Forward	5′-TACTGCCGGAGTCTGACTGGA-3′	86
		Reverse	5′-TCTGTCTCAATACCGCAGTCCA-3′	
*GAPDH*	NM_017008.4	Forward	5′-TCACCATCTTCCAGGAGCGA-3′	102
		Reverse	5′-CCTTCTCCATGGTGGTGAAGA-3′	
